# Vegetation Structure and Environmental Correlates of Climbing Behavior for Desert Shrub *Ochradenus baccatus*

**DOI:** 10.3390/plants14111696

**Published:** 2025-06-01

**Authors:** Dhafer A. Al-Bakre

**Affiliations:** Department of Biology, College of Science, University of Tabuk, Tabuk 71491, Saudi Arabia; dalbakre@ut.edu.sa

**Keywords:** growth form, desert plant, environmental gradients, indicator species, microhabitats, distribution

## Abstract

*Ochradenus baccatus* Delile (Resedaceae) is a widely distributed desert shrub known for its remarkable growth form plasticity, growing either independently or as a facultative climber on other vegetation. Despite its ecological adaptability, the drivers underlying its dual growth strategy remain poorly understood in arid ecosystems. This study aimed to investigate the growth form plasticity of *O. baccatus* across diverse ecological gradients in Saudi Arabia and identify key environmental and floristic factors influencing its climbing and independent forms. Field surveys were conducted from 2020 to 2024 across 103 sites, using stratified random sampling. At each site, vegetation data were collected using 50 × 50 m quadrats, and species composition, life form percentage, and *O. baccatus* behavior were recorded. Results revealed clear ecological separation between behaviors. Climbing individuals were associated with higher elevations, greater tree and shrub cover, and moderate soil fertility, while independent individuals were broadly distributed in herbaceous and open habitats. Diversity indices (Shannon, Simpson, evenness) increased with altitude, particularly in climbing habitats. PERMANOVA confirmed significant differences in species composition between behaviors (*p* = 0.0001), and SIMPER analysis identified species like *Haloxylon salicornicum* and *Zygophyllum album* as key contributors in climbing habitats. Indicator species analysis revealed behavior-specific taxa, while CCA demonstrated that rainfall, soil moisture, and temperature were the strongest environmental predictors of growth behavior. This study highlights the ecological flexibility of *O. baccatus* and the role of environmental filtering and plant community structure in shaping its growth strategy. These results have implications for the growth form plasticity of desert plants and can be applied to vegetation management and restoration in arid ecosystems.

## 1. Introduction

Desert ecosystems, characterized by arid and hyperarid climates, host an unexpectedly high level of biodiversity despite extreme environmental conditions [1]. Desert vegetation tends to exhibit high endemism and ecological specialization, indicated by long-term adaptation to stringent climatic and edaphic conditions [2]. However, in recent decades, desert biodiversity has become increasingly susceptible to various disturbances, including habitat degradation, overgrazing, climate fluctuation, and anthropogenic impacts such as urban expansion, off-road vehicle activity, and infrastructure development [3]. Knowledge about the adaptive strategies adopted by native vegetation in such stressful environments is of prime importance not only for biodiversity conservation but also for developing ecological restoration and sustainable land management techniques in arid lands [4]. In xeric conditions, plant species have developed a variety of morphological and behavioral adaptations to counteract the adversity of water limitation [5]. Among these, plasticity in growth form, where plants change their structural growth, such as taking up climbing or autonomous growth forms by environmental stimuli, is notably important [6]. Such plasticity in behavior enhances the survival of plants in spatially and temporally variable microhabitats by allowing response to particular environmental conditions like soil water content, variability in rainfall, temperature gradients, and proximity to structurally supportive vegetation, all of which impact the expression of plant growth forms [7]. The environmental drivers and floristic associations that drive growth form variation in desert plants are still not well understood [8]. Filling these knowledge gaps is crucial to increasing our understanding of species/environment interactions and the functional diversity that maintains desert ecosystems [9].

Keystone desert plants function as ecological engineers, holding soil in place, regulating microclimates, and maintaining complex biological communities [10]. As a rule, they become key elements in sparse desert vegetation networks, affecting resource distribution and ecosystem functioning [11]. Their survival strategies for harsh conditions lead them to the center of ecological research, conservation planning, and habitat restoration programs [12]. *Ochradenus baccatus* Delile (Resedaceae) is a common semi-deciduous shrub dominant in the Arabian Peninsula, North Africa, and the Middle East [13,14]. In Saudi Arabia, it encroaches on a broad spectrum of diverse habitats ranging from rocky hill slopes and stony wadis to sandy plains [15]. The species is highly valued for its ecological functions, ranging from soil stabilization, protection of small animals, and nurse planting in degraded habitats [16]. Beyond ecological significance, *O. baccatus* also has high medicinal potential because of its broad profile of secondary metabolites whose antioxidant, antibacterial, and antiparasitic activities have been reported [17]. Its economic and ecological significance position *O. baccatus* as a model species for understanding plant survival strategies in arid ecosystems [18].

Among *O. baccatus*’s typical ecological features is that it has plasticity characteristics so that it could either exist autonomously as a self-sufficient shrub or as a facultative climber by leaning over other vegetation that support it [19]. This adaptive characteristic of growth form plasticity is believed to be environmentally motivated, with adaptations in habitat architecture, soil health, and watering levels inducing responses [20]. Despite its ecological importance and widespread presence across Saudi Arabia, the determinants of climbing versus independent growth behaviors in *O. baccatus* remain poorly understood. Of particular concern is a significant knowledge gap regarding how local environmental gradients influence its growth strategies across highly heterogeneous desert environments. The current study was initiated with the following objectives: to explore the growth form plasticity of *O. baccatus* based on comparisons of its climbing and independent growth habits under different ecological conditions in Saudi Arabia; to analyze the floristic composition and life form structure related to each growth habit and identify indicator species typical of their respective environments; and to evaluate the effect of environmental factors on the distribution and ecological expression of climbing and independent growth strategies. To attain these objectives, the research solves three core research questions: (i) How and why do climbing and self-supporting growth forms of *O. baccatus* differ between different ecological environments?, (ii) What are the floristic and structural differences between the habitats of each growth form, and which plant species are good indicators of these habits?, and (iii) Which environmental factors best predict the presence and spatial abundance of climbing versus self-supporting strategies?

## 2. Results

### 2.1. Associated Floristic Composition

One hundred thirty-nine plant species were recorded in association with *O. baccatus* exhibiting both climbing and independent growth behaviors. These species spanned a diverse range of plant families, with Asteraceae (20 species), Fabaceae (15 species), Amaranthaceae (12 species), and Poaceae (12 species) being the most represented. Other notable families included Apocynaceae (8 species), reflecting the ecological diversity of associated flora. Interestingly, 26 families were represented by either a single species (21 families) or two species (5 families), indicating a high proportion of monospecific associations. This pattern highlights a mix of generalist and specialist interactions, suggesting that *O. baccatus* occupies a structurally and compositionally varied ecological niche across its range in Saudi Arabia.

### 2.2. Structure and Habitat Differentiation

**Figure 1:**
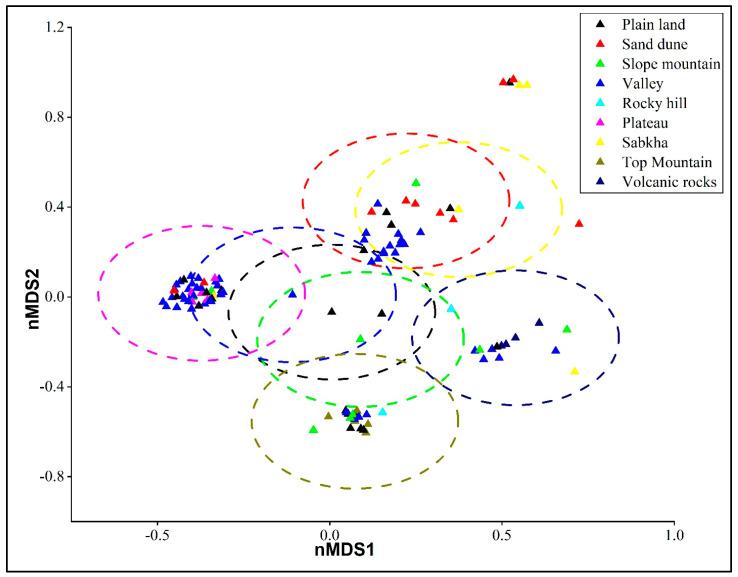
Non-metric multidimensional scaling (NMDS) ordination of species composition across different habitat types associated with *O. baccatus* in Saudi Arabia. Each point represents a sampling plot, and colors/symbols indicate distinct habitat types. Dashed ellipses represent 95% confidence intervals around habitat group centroids, highlighting differences in community structure.

Non-metric multidimensional scaling (NMDS) ordinations revealed clear patterns in species composition across different habitat types, with samples clustering according to habitat (Figure 1). The PERMANOVA test confirmed statistically significant differences in community composition across habitats (F = 2.892, *p* = 0.0001, permutations = 9999), indicating that habitat type has a strong influence on the distribution of species associated with *O. baccatus*. The total sum of squares was 33.94, with within-group variation accounting for 27.24, supporting the observed between-habitat variation. SIMPER (Similarity Percentage) analysis was used as an exploratory tool to identify species contributing most to compositional dissimilarities among habitats. *Haloxylon salicornicum, Peganum harmala, Ochradenus baccatus, Lycium shawii*, and *Vachellia gerrardii* were among the species most frequently associated with variation in species composition, collectively accounting for over 12.5% of the observed dissimilarity (Supplementary Materials data). These species showed marked variation in abundance across different habitats, highlighting their role in distinguishing ecological communities. For example, *Peganum harmala* was highly abundant in rocky hills, while *O. baccatus* showed a more evenly distributed presence but was particularly prominent in volcanic rock and mountain top habitats.

### 2.3. Influence of Altitude on Diversity Indices by Growth Behavior

**Figure 2:**
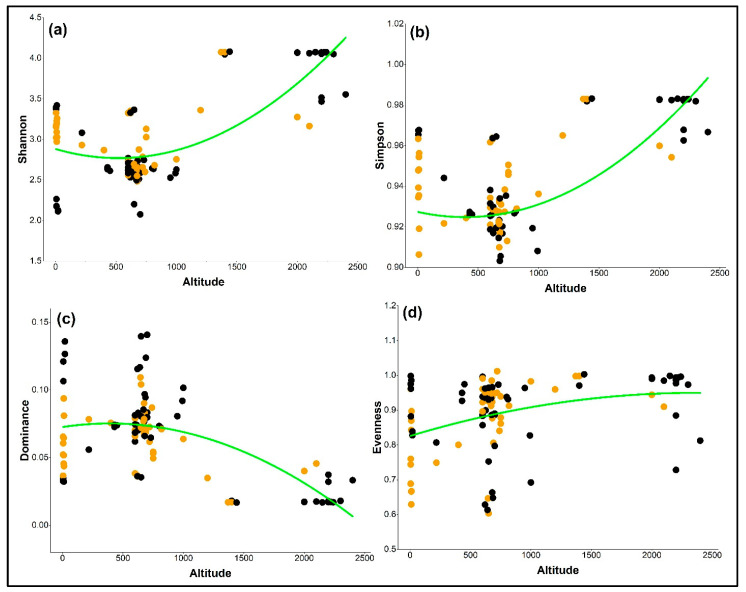
Polynomial relationships between altitude and plant diversity indices associated with climbing (orange) and independent (black) growth behaviors of *O. baccatus* across sampled habitats in Saudi Arabia. (**a**) Shannon index, (**b**) Simpson index, (**c**) dominance, and (**d**) evenness. Green lines represent fitted second-degree polynomial regression curves. Climbing individuals tend to show higher diversity and evenness and lower dominance at higher elevations compared to independent individuals.

Analysis of diversity indices along an altitudinal gradient showed distinct and behavior-specific trends within plant communities related to *O. baccatus*. The Shannon index, which measures both species richness and evenness, and the Simpson index, which places greater emphasis on species dominance, were utilized to measure complementary elements of community diversity. The Shannon and Simpson diversity indices exhibited significant positive quadratic relationships with altitude, with coefficients of determination R^2^ = 0.376 and R^2^ = 0.399, respectively (Figure 2a,b). These results indicate increasing species richness and diversity at higher elevations. This pattern was especially evident in climbing individuals, which consistently showed higher Shannon and Simpson values at middle to high altitudes (>1500 m). The findings suggest that climbing forms of *O. baccatus* may support or benefit from more structurally complex and species-rich communities in elevated habitats, possibly due to more stable microclimates and host availability.

Conversely, dominance values declined significantly with altitude (R^2^ = 0.418; Figure 2c), reflecting a reduced influence of a few dominant species in highland environments. This trend was more prominent in climbing behavior, where lower dominance values were observed consistently across elevation gradients. This likely indicates a shift toward more balanced species interactions in diverse highland ecosystems. Evenness showed a moderate increase along the altitudinal gradient (R^2^ = 0.193; Figure 2d), with climbing-associated plots generally exhibiting more uniform species distributions compared to independent ones. Although the explained variance was lower than for other indices, this pattern still points to improved community structure and reduced species skewness at higher elevations (Table 1).

### 2.4. Association of Growth Behavior with Vegetation Structure and Altitude

**Figure 3:**
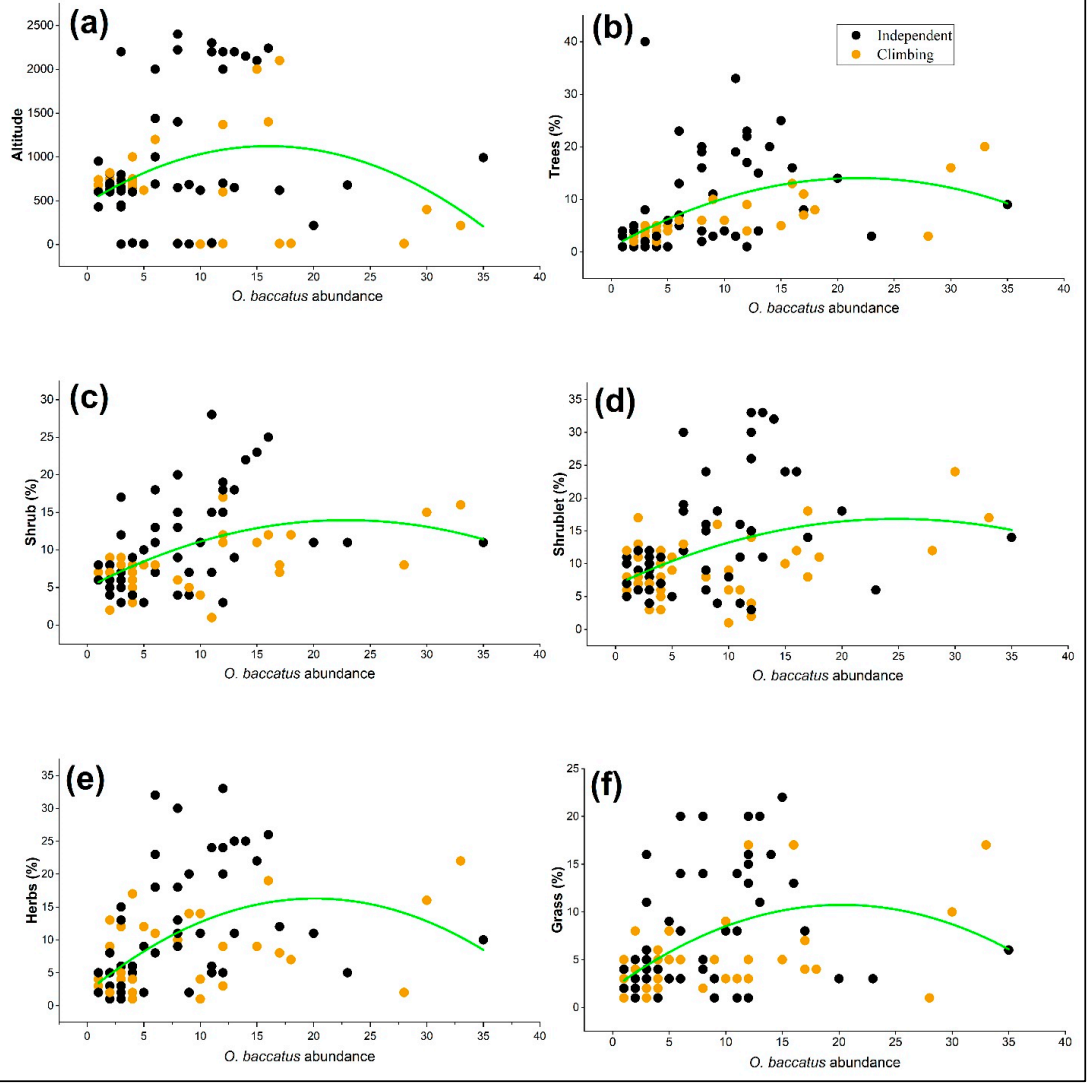
Relationships between the abundance of *O. baccatus* and ecological variables across sampled sites, separated by growth behavior: independent (black points) and climbing (orange points). Polynomial regression curves (green) illustrate trends for (**a**) altitude, (**b**) tree cover percentage, (**c**) shrub cover percentage, (**d**) shrublet cover percentage, (**e**) herb cover percentage, and (**f**) grass cover percentage.

The abundance and growth behavior of *O. baccatus* varied significantly in response to altitude and the composition of surrounding vegetation life forms (Figure 3). Polynomial regression analyses revealed that *O. baccatus* abundance showed a weak but significant quadratic relationship with altitude (R^2^ = 0.097, *p* = 0.0015), peaking at mid-elevations (~1000–1500 m) and declining toward both lower and higher extremes. This pattern was more evident in climbing individuals, which were largely concentrated in mid- to high-altitude areas. Climbing individuals (orange points) were more frequently associated with sites rich in trees and tall shrubs. This is supported by significant positive relationships between *O. baccatus* abundance and tree (R^2^ = 0.248, *p* < 0.0001) and shrub (R^2^ = 0.248, *p* < 0.0001) cover. In contrast, independently growing individuals were more broadly distributed across the gradient and occurred more commonly in communities dominated by herbs (R^2^ = 0.275, *p* < 0.0001), grasses (R^2^ = 0.209, *p* < 0.0001), and shrublets (R^2^ = 0.167, *p* = 0.0015) (Table 2). These behavior-specific patterns indicate that *O. baccatus* displays growth form plasticity, adapting its growth strategy based on available vegetation structure. Climbing behavior is closely linked to woody life forms and higher elevations, while independent individuals dominate herbaceous, open, and possibly more disturbed habitats.

### 2.5. Environmental Drivers of Growth Behavior

**Figure 4:**
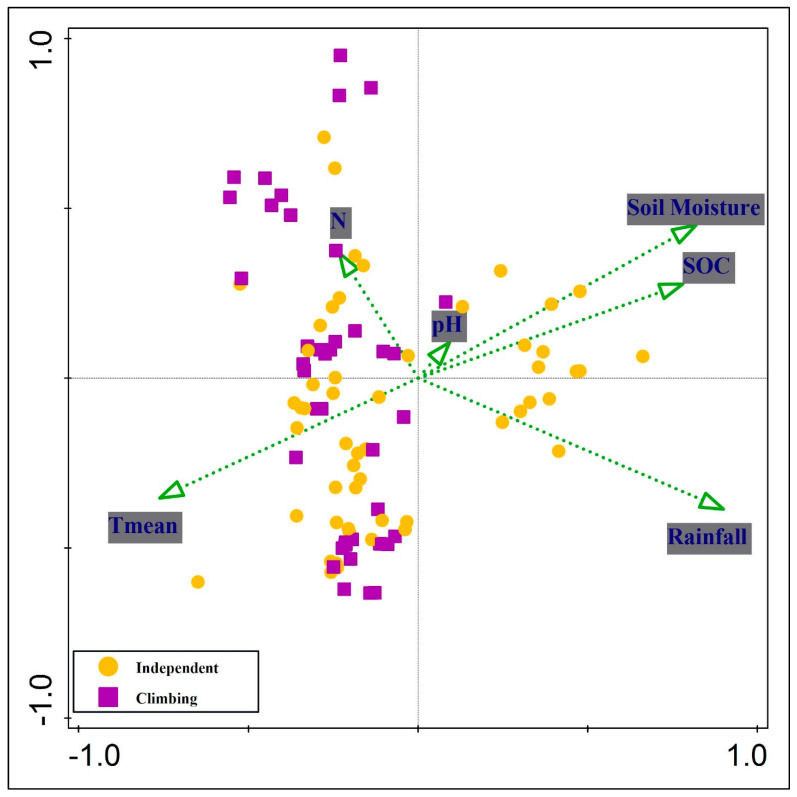
Canonical Correspondence Analysis (CCA) ordination plot showing the distribution of *O. baccatus* growth behaviors—climbing (purple squares) and independent (orange circles)—in relation to environmental variables. Green arrows represent significant environmental gradients, including rainfall, soil moisture, mean annual temperature (Tmean), soil organic carbon (SOC), nitrogen (N), and pH. Climbing individuals are associated with higher soil moisture, SOC, and rainfall, while independent individuals are more dispersed and tend to occur under drier or less nutrient-rich conditions.

CCA revealed significant separation in the distribution of *O. baccatus* growth behaviors—climbing and independent—in relation to key edaphic and climatic variables (Figure 4). The analysis accounted for a total constrained variation of 4.578, with environmental predictors explaining 31.9% of the total variation (adjusted explained variation: 27.6%). The first two CCA axes were especially informative, with eigenvalues of 0.6448 (Axis 1) and 0.4849 (Axis 2), together explaining 24.68% of the constrained variation. The cumulative explained fitted variation reached 44.18% on Axis 1 and 77.4% on Axis 2 (Table 3). The pseudo-canonical correlations for these axes were also high—0.8918 and 0.8336—indicating strong relationships between species distributions and environmental gradients. Additional axes (Axes 3 and 4) had eigenvalues of 0.2527 and 0.0588, with the cumulative explained fitted variation reaching 94.72% and 98.75%, respectively.

Simple term effects revealed that rainfall (13%), soil moisture (11.8%), mean temperature (11%), and soil organic carbon (9.6%) were the most significant variables structuring the behavior of *O. baccatus* (*p* = 0.002 for all), while nitrogen also contributed significantly (3.6%, *p* = 0.012). Soil pH had no significant effect (*p* = 0.74). Conditional term effects confirmed rainfall (13%) and soil moisture (10%) as the strongest independent predictors, followed by mean temperature (5.4%, *p* = 0.002) and nitrogen (2.1%, *p* = 0.048) (Table 4).

### 2.6. Indicator Species Associated with Both Behavior

Indicator species analysis was employed as an exploratory method to detect taxa that were always related to the climbing or independent growth behaviors of *O. baccatus*. Of the species that were more often found in environments dominated by climbing, *Haloxylon salicornicum* and *Zygophyllum album* had the greatest indicator values (IndVal% = 3.35 and 3.58, respectively; *p* < 0.05), indicating a persistent occurrence in those habitats. Other taxa with high indicator values for climbing habit were *Salsola rosmarinus*, *Panicum turgidum*, *Zilla spinosa*, and *Salsola foetida*, which were all species that are typically encountered in arid, shrub-dominated environments. For independently growing plants, taxa like *Aerva javanica*, *Boerhavia repens*, *Indigofera spinosa*, *Vachellia flava*, and *Lycium shawii* were found to have comparatively high indicator values. Whereas *Aerva javanica* was one of the most regularly linked to independent types (IndVal% = 2.196, *p* = 0.0281), such associations must be regarded as co-occurrence patterns and not necessarily ecological causation (Table 5).

**Figure 5:**
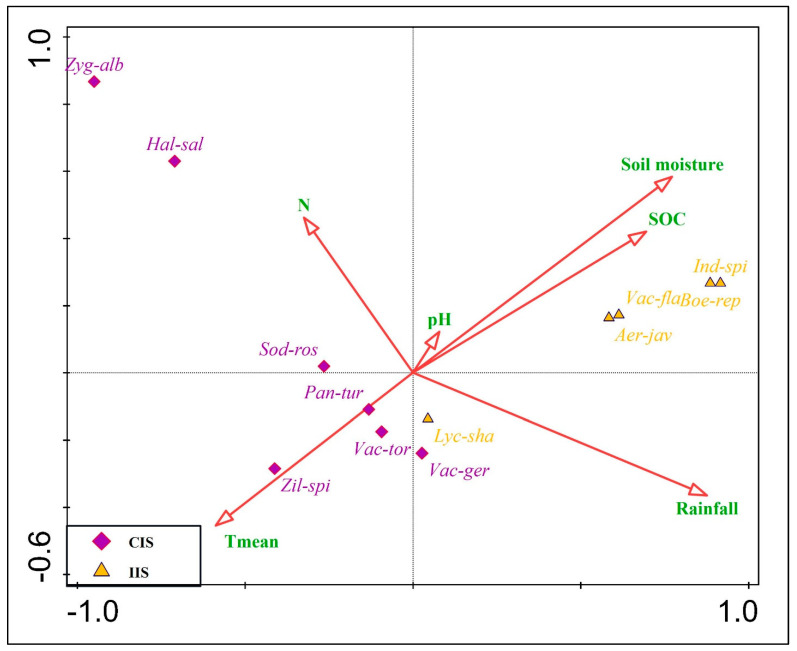
Canonical Correspondence Analysis (CCA) ordination plot showing the relationship between indicator species and environmental gradients associated with climbing (CIS, purple diamonds) and independent (IIS, yellow triangles) growth behaviors of *O. baccatus*. Species codes represent the following taxa: Zyg-alb = *Zygophyllum album*, Hal-sal = *Haloxylon salicornicum*, Sod-ros = *Salsola rosmarinus*, Pan-tur = *Panicum turgidum*, Zil-spi = *Zilla spinosa*, Vac-tor = *Vachellia tortilis*, Vac-ger = *Vachellia gerrardi*, Lyc-sha = *Lycium shawii*, Aer-jav = *Aerva javanica*, Boe-rep = *Boerhavia repens*, Vac-fla = *Vachellia flava*, Ind-spi = *Indigofera spinosa*. Red arrows indicate significant environmental gradients: mean annual temperature (Tmean), nitrogen (N), soil pH, soil organic carbon (SOC), soil moisture, and rainfall. Climbing species are generally aligned with temperature and nitrogen, while independent species are strongly associated with higher rainfall, soil moisture, and organic content.

CCA further clarified these associations. Climbing indicator species were positioned on the left side of the ordination, correlating with gradients of mean annual temperature (Tmean) and soil nitrogen (N), suggesting adaptation to warmer, nutrient-moderate environments. In contrast, independent indicator species were aligned with rainfall, soil organic carbon (SOC), and soil moisture (Figure 5). The positioning of *Haloxylon salicornicum* and *Zygophyllum album* furthest from the origin underscores their strong specialization and association with climbing behavior in arid, xeric conditions. Meanwhile, *Aerva javanica, Boerhavia repens*, and *Indigofera spinosa* grouped closely in the direction of high moisture and SOC, consistent with their role as indicators of independent *O. baccatus* growth in more favorable soil conditions.

## 3. Discussion

The elevated floristic diversity documented in conjunction with *O. baccatus* corroborates the species’ habitat tolerance and wide ecological adaptability, consistent with earlier findings that desert shrubs tend to serve as keystone species in arid vegetation communities [21,22]. The dominance of Asteraceae, Fabaceae, Amaranthaceae, and Poaceae echoes trends within other arid environments, where they exhibit strong adaptive radiation under water limitation [23,24]. The occurrence of numerous monospecific families is indicative of the double-sided nature of *O. baccatus* interactions, both with generalist species and specialists of abiotic microhabitat adaptations [25]. This combination of generalist and specialist membership is consistent with desert plant communities research, whereby structurally elaborate areas harbor widespread and narrowly specialized taxa [26,27]. The wide taxonomic memberships of *O. baccatus* are also indicative of its behavioral flexibility, allowing it to utilize different vegetation structures using independent and climbing forms. These results corroborate the hypothesis that facilitative interactions and niche plasticity are central survival tactics in deserts, where resource heterogeneity governs community dynamics [28]. The wide variety of associated plant families emphasizes the functional significance of *O. baccatus* in preserving ecological complexity in xeric ecosystems.

The distinctive assemblage of species among habitats in conjunction with *O. baccatus* underlines the role of environmental heterogeneity in contributing to desert plant community structure. The patterns of clustering and differences in habitats align with ecological principles that focus on microhabitat variability as the primary force influencing species assembly in arid environments [29,30]. The recognition of *Haloxylon salicornicum*, *Peganum harmala*, *Lycium shawii*, and *Vachellia gerrardii* as important players in habitat differences reinforces previous research that emphasizes their contributions to desert vegetation mosaics [31,32]. Their extreme habitat affinities, such as *Peganum harmala* on rocks and *Haloxylon salicornicum* on stabilized sand, confirm the connection between substrate type and species distribution [33]. *O. baccatus*‘s wide but habitat-conforming range across volcanic rocks and mountain slopes, reflecting its ecological flexibility, allows it to survive under varying edaphic and climatic pressures. These findings are consistent with past studies proving that multifunctional shrubs are structural keystones of arid ecosystems, facilitating biodiversity through multiple interactions [34,35]. The synchronized species abundance variation also emphasizes habitat differentiation in desert community diversity and resilience [36].

The altitudinal gradient in diversity indices captures the influence of elevation on plant community structure and species interactions in desert communities. The increase in Shannon and Simpson diversity with elevation is in line with previous literature, suggesting that more stable microclimates, greater moisture, and greater structural complexity at higher elevations enable more diverse species [37,38]. The more vigorous response in climbing plants indicates that facilitative vegetation and more moderate conditions at higher elevations support more diverse, stratified plant communities and enable facultative climbing [39,40]. The reduction in dominance with altitude also suggests that highland habitats sustain more balanced interactions among species, minimizing the effect of short-dominant taxa evident in more extreme lowlands [41]. The shift towards higher evenness between climbing-related plots also indicates higher niche availability and more balanced resource distribution in structurally complex environments [42].

The variation in growth behavior and density of *O. baccatus* along altitudinal and vegetation gradients underlines its ecological plasticity and resilience in heterogeneous desert environments. Mid-elevation peak in climbing individuals translates into larger-scale patterns where mid-elevations provide optimal microhabitats with well-balanced moisture, temperature, and structural support [43,44]. The close association between climbing behavior and higher tree and tall shrub cover confirms that physical support and canopy complexity are major drivers of facultative climbing in desert habitats [45,46]. The same trends in xeric habitats indicate that vertical vegetation structure not only offers mechanical support but also enhances microhabitat conditions, favoring climbing forms [47]. The broader occupation of open habitats and herbaceous environments by autonomous individuals represents an overall generalist survival strategy to allow persistence within less structured, and possibly disturbed, environments [48]. Their occurrence with herbs and grass-dominated communities suggests that in the absence of provision of structure, *O. baccatus* assumes a free-standing morphology, consistent with behavioral plasticity as the primary survival strategy [49]. These findings highlight that vegetation structure and structural availability are strong ecological filters that control *O. baccatus* growth behavior and its broad ecological range in desert ecosystems.

The climatic and edaphic conditions significantly effect in shaping of *O. baccatus* behavioral tactics in arid environments. The preference of climbing plants for rainier habitats, moist soils, and organic carbon content as seen in the field highlights the ecological importance of resource-rich microhabitats in the realization of structural growth flexibility [50]. Far from reacting passively to their environment, such plants *as O. baccatus* seem to capitalize on beneficial abiotic conditions by assuming growth forms favoring greater access to light and vertical space. This concords with mature ecological theory proposing that greater availability of resources enhances community complexity and niche divergence [46,51]. As compared to this, the predilection for lone plants to excel in drier, nutrient-limited environments is indicative of a conservative mode of growth adaptive to more hostile environments with reduced structural support [29,42]. Such disparate responses serve to underscore the functional importance of growth form plasticity in optimizing survival over heterogeneous landscapes [52]. In addition, the limited effect of soil pH supports evidence from arid ecosystem ecology, where gradients of moisture and nutrients and not chemical soil factors increasingly determine species patterns [53,54,55]. These trends highlight the importance of environmental filtering in determining the spatial distribution of plastic growth patterns in *O. baccatus*.

The detection of specific indicator species associated with the climbing and independent lifestyles of *O. baccatus* highlights the significance of vegetation structure, community composition, and environmental gradients in the formation of behavioral strategies in desert ecosystems [10]. The intensive linking of climber individuals to species like *Haloxylon salicornicum* and *Zygophyllum album* fits the notion that facultative climbing is favored within tall, woody plant-dominant environments with the capability of mechanical support [56]. Other climbing signs, such as *Salsola rosmarinus*, *Panicum turgidum*, and *Zilla spinosa*, support the idea that the vertical structuring of the plant community is a major facilitator of climbing behavior [57]. Conversely, autonomous plants were predominantly linked with herbaceous and low shrubby plants such as *Aerva javanica*, *Boerhavia repens*, and *Indigofera spinosa*, plants generally hegemonic in open and less structurally rich environments, where independent, free-standing development is an imperative adaptive solution [58,59]. Climbing-associated taxa were closely related to mean annual temperature and soil nitrogen gradients, indicating climbing behavior is favored under relatively warmer conditions where structural support exists [60]. The leading placement of *Haloxylon salicornicum* and *Zygophyllum album* in the ordination indicates their strong specialization toward xeric, drier environments but within communities that provide adequate vertical architecture [61,62]. Conversely, species associated with independence were grouped with greater rainfall, soil organic carbon, and soil moisture gradients, reflecting a preference for more fertile, mesic conditions that support herbaceous communities [63]. Clustering of species such as *Aerva javanica* and *Boerhavia repens* on these gradients implies that independent foraging by *O. baccatus* is especially advantageous in wet, organically nutrient-rich environments where competition for vertical space is avoided [64].

The findings of this study have important ecological and conservation implications for plant behavioral adaptations and desert ecosystem management. The demonstrated behavioral plasticity of *O. baccatus* in vegetation structure, topographic heterogeneity, and environmental gradients highlights the key role of habitat heterogeneity in sustaining functional biodiversity in arid ecosystems. Knowledge that structurally intricate, water-dense environments foster climbing behavior and solitary growth dominates while more open, resource-scarce habitats are valuable for habitat rehabilitation and restoration strategies. In disturbed desert environments, encouraging the reestablishment of woody species that create vertical structure may increase the survival and ecological role of behaviorally plastic species like *O. baccatus*. In addition, the recognition of behavior-specific indicator species allows for a pragmatic approach to tracking ecosystem health and vegetation dynamics in the context of climatic fluctuations. As arid ecosystems become more vulnerable to climatic shift and human encroachment, it is vital to understand the interactions between plant behavior, community, and the environment to predict changes in vegetation, retain biodiversity, and develop adaptive management strategies that support ecosystem resilience.

## 4. Materials and Methods

### 4.1. Study Area

**Figure 6:**
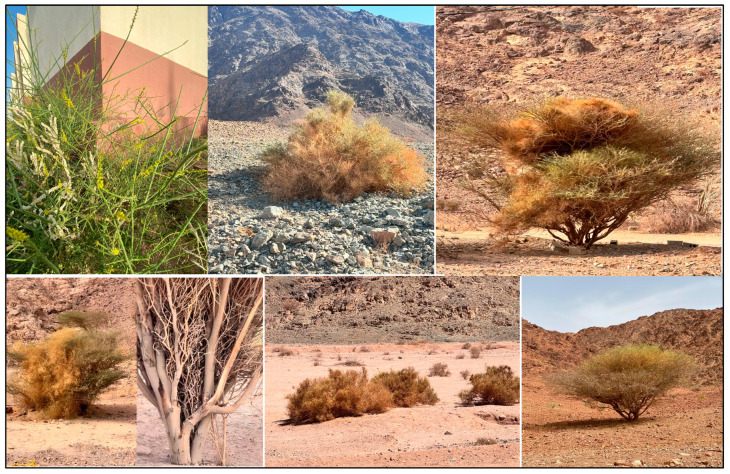
Field images illustrating the morphology and behavioral growth forms of *O. baccatus* in various habitats across Saudi Arabia.

*O. baccatus* has a gynodioecious breeding system, with female and hermaphroditic forms, and shows bimodal spring and autumn flowering maxima that allow adaptation to changing desert environments. Ecologically, *O. baccatus* is exceptionally behaviorally plastic, occurring as a solitary shrub or facultative climber depending on local habitat conditions (Figure 6). Climbers are normally found in rough, vegetated environments, whereas solitary forms predominate in more open, xeric sites.

**Figure 7:**
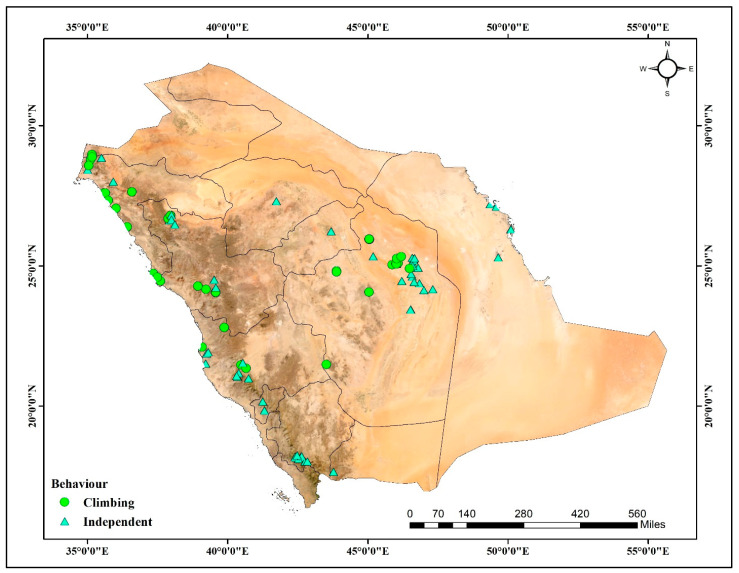
Geographic distribution of *O. baccatus* growth behaviors across Saudi Arabia. Green circles represent climbing individuals, while cyan triangles denote independent individuals. Climbing forms are concentrated along the mountainous western escarpments of the Arabian Shield, whereas independent forms show a broader distribution across central and eastern regions, including plains, plateaus, and sandy deserts.

This study was conducted across a broad ecological gradient in Saudi Arabia, a country marked by its geomorphological duality: the Precambrian Arabian Shield in the west and the Phanerozoic Arabian Shelf in the east [44]. The region is characterized by high elevation, rugged terrain, volcanic fields, and narrow valleys, while the shelf features vast plateaus, sedimentary plains, and low-relief desert basins [65]. Soil types vary accordingly, with silt loam dominating the elevated western escarpments, sandy loam prevalent in eastern lowlands, and loam concentrated in central wadis and valleys [66]. Vegetation cover is sparse and patchy due to aridity but is densest in the southwestern highlands and along drainage networks where moisture and deeper soils persist [67]. *O. baccatus* was found distributed across the western, central, and eastern regions (Figure 7). Climbing individuals were concentrated in the higher-elevation, moisture-retentive terrains of the Arabian Shield, while independent forms exhibited wider ecological tolerance, extending into the sandy plains and gravel deserts of the Arabian Shelf.

### 4.2. Field Survey and Vegetation Sampling

To evaluate the ecological distribution, life form association, and growth form plasticity of behavior of *O. baccatus* over a variety of habitats in Saudi Arabia, an extensive field survey was carried out for four years, from 2020 to 2024. The work included 103 field sites chosen to represent as wide a selection of environmental gradients as possible within the Arabian Peninsula, including the Arabian Shield and Arabian Shelf. Sites were divided into categories depending on growth behavior of *O. baccatus*: 56 sites had independent individuals, whereas 47 sites favored climbing individuals, frequently growing on or supported by Vachellia species and other woody hosts. Vegetation data were recorded at each site by the quadrat sampling method, a standard procedure for community-level plant measurement. Four 50 × 50 m quadrats (500 m^2^) were set per site, being stratified-randomly to mirror local geomorphological diversity (e.g., slope, elevation, and landform position) [68]. All the species of vascular plants present in each quadrat were recorded, and the number of individuals of each species was counted to assess abundance (individuals per 500 m^2^). Species were also grouped according to growth form—trees, shrubs, shrublets, herbs, and grasses—and their respective percentage cover was estimated.

Canopy cover was determined using a crown projection method based on average canopy diameter [69], applying the formula:

(1) $$A=\pi r^{2}$$

where *r* is the mean canopy radius. The cumulative area of canopy per species was used to calculate total and relative cover within each quadrat. From these data, density, frequency, and relative abundance were calculated, enabling comparisons between sites and behaviors [70].

### 4.3. Environmental Data Acquisition

To complement field-based vegetation surveys, environmental variables were extracted using geospatial datasets and tools to characterize the abiotic conditions of each site. For each of the 103 sites surveyed, GPS coordinates were recorded in the field using handheld GPS units. These coordinates served as the spatial reference for retrieving climate and soil data using ArcGIS Pro (version 10.2). Climatic variables, including mean annual temperature (Tmean) and annual precipitation (rainfall), were obtained from the WorldClim v2 database, which provides high-resolution (~1 km^2^) global climate layers based on 1970–2000 long-term averages. These raster layers were imported into ArcGIS Pro, and zonal statistics were used to extract climate values for each sampling point. To capture soil characteristics, high-resolution soil property maps from the SoilGrids database (ISRIC—World Soil Information) were used. Soil variables were extracted for the 0–15 cm depth layer and included soil moisture (%), soil organic carbon (SOC, g/kg), total nitrogen (N, g/kg), and pH (measured in H_2_O). Spatial layers were overlaid with the site coordinates, and attribute values were extracted directly using the “Extract Multi Values to Points” tool in ArcGIS Pro.

### 4.4. Data Analaysis

A combination of multivariate, univariate, and ordination techniques was applied to analyze the distribution patterns, ecological associations, and environmental drivers of the two growth behaviors (climbing vs. independent) of Ochradenus baccatus across various habitats in Saudi Arabia. Analyses were conducted using R software (version 4. Aukland, New Zealand), PAST (version 4.11, Oslo, Norway), OriginPro 2023 (Northampton, MA, USA), and CANOCO 5.0 (Ithaca, NY, USA). Community composition was assessed using non-metric multidimensional scaling (NMDS) based on Bray–Curtis dissimilarity matrices, implemented through the metaMDS function in the vegan package in R [71]. Differences in species composition among habitat types and between behaviors were statistically tested using PERMANOVA (Permutational Multivariate Analysis of Variance) with 9999 permutations via the adonis function. To identify which species contributed most to group dissimilarity, Similarity Percentage (SIMPER) analysis was also performed using the simper function.

To identify indicator species that significantly differentiate between climbing and independent behaviors, indicator species analysis was conducted using the multipatt function in the indicspecies package in R [72]. This analysis provided indicator values (IndVal%) and associated *p*-values through permutation tests (999 permutations), highlighting taxa with strong fidelity and specificity to either behavior type. Species diversity across sampling sites was assessed using four commonly used indices: Shannon (H’), Simpson (1-D), dominance (D), and evenness (E), all computed using PAST software [73]. These diversity indices were then related to elevation using second-degree polynomial regression models in OriginPro, following the general equation:

(2) $$y=\beta_{0}+\beta_{1}x+\beta_{2}\beta x^{2}$$

where *y* is the diversity index and *x* is altitude. Regression coefficients, R^2^ values, and *p*-values were used to assess the strength and significance of these relationships.

Second-degree polynomial regression was used to fit the relationship between *O. baccatus* abundance and percentage cover of important life-form groups because this form of model was desired to capture possible non-linear ecological responses, i.e., mid-range peaks or decreases in abundance along structural gradients, which are typical in variable desert environments. These analyses, visualized in Figure 5, revealed distinct behavior-specific patterns: climbing individuals were more closely associated with higher altitudes and woody vegetation, whereas independent individuals were more frequently found in herbaceous and open environments. To explore the role of environmental factors in shaping species composition and growth behavior, Canonical Correspondence Analysis (CCA) was conducted using CANOCO 5.0 [74]. The constrained ordination included soil properties and climatic variables. Output included eigenvalues, pseudo-canonical correlations, and explained variation across the first four axes. The significance of environmental variables was assessed through simple and conditional term effects using Monte Carlo permutation tests (999 permutations).

## 5. Conclusions

This research presents unequivocal evidence that behavioral plasticity in *O. baccatus* is highly controlled by environmental gradients and vegetation structure throughout Saudi Arabia’s desert landscapes. Climbing forms preferentially inhabit wet, rich, and structurally heterogeneous environments at middle to high elevations, in which tree and tall shrub cover supports vertical growth. Independent forms, however, prevail in herbaceous and open habitats with lower moisture availability and lower vegetation architecture. Environmental drivers such as mean temperature, rainfall, and soil moisture were found to be the most important drivers of growth behavior. Indicator species analysis also indicated that some plant assemblages are associated with each of the two growth behaviors, suggesting niche differentiation. Results stress habitat heterogeneity and resource access as the principal determinants of behavioral responses among desert plants and emphasize the functional role of *O. baccatus* in enhancing arid vegetation networks. Implications from the findings of this work improve the scope of understanding concerning plant adaptive systems and are immensely important in contributions to vegetation planning and rehabilitation under increasingly stressful arid environments.

## Figures and Tables

**Table 1:** Summary of polynomial regression analysis between altitude and diversity indices associated with *O. baccatus* growth behaviors. The table includes the coefficient of determination (R^2^), slope of the linear term (B1), and significance level (*p*-value) for each diversity metric. All relationships are based on second-degree polynomial models.

Diversity Index	Linear Coefficient	SE (±)	R^2^	*p*-Value
**Shannon**	−0.000436	0.00019	0.376	<0.001
**Simpson**	−0.000013	1.30 × 10^−5^	0.399	<0.0001
**Dominance**	−0.000028	1.30 × 10^−5^	0.418	<0.0001
**Evenness**	0.000031	1.20 × 10^−5^	0.193	<0.001

**Table 2:** Summary of polynomial regression analysis showing the relationship between *O. baccatus* abundance and key ecological variables, including altitude and percentage cover of various plant life forms. The table presents the linear coefficient (B1), standard error (±), coefficient of determination (R^2^), and significance level (*p*-value) for each predictor. All models are second-degree polynomial fits.

Variable	Linear Coefficient	SE (±)	R^2^	*p*-Value
**Altitude**	81.16364	24.78134	0.09694	<0.001
**Trees (%)**	1.20723	0.25785	0.24774	<0.0001
**Shrub (%)**	0.7897	0.1755	0.24784	<0.0001
**Shrublet (%)**	0.81054	0.24799	0.16692	<0.001
**Herbs (%)**	1.41565	0.2623	0.27492	<0.0001
**Grass (%)**	0.86468	0.19302	0.20916	<0.0001

**Table 3:** Canonical Correspondence Analysis (CCA) axis summary statistics for the relationship between *O. baccatus* growth behaviors and environmental variables. The table includes eigenvalues, explained variation, pseudo-canonical correlations, and cumulative explained fitted variation for the first four CCA axes.

Statistic	Axis 1	Axis 2	Axis 3	Axis 4
**Eigenvalues**	0.6448	0.4849	0.2527	0.0588
**Explained variation (cumulative)**	14.08	24.68	30.2	31.48
**Pseudo-canonical correlation**	0.8918	0.8336	0.6011	0.524
**Explained fitted variation (cumulative)**	44.18	77.4	94.72	98.75

**Table 4:** Summary of simple and conditional effects from CCA evaluating the influence of environmental variables on the distribution of climbing and independent behaviors of *O. baccatus*. Presented values include the percentage of variation explained, pseudo-F values, and associated *p*-values for each variable.

Variable	Simple Effect (%)	Simple Pseudo-F	Simple *p*-Value	Conditional Effect (%)	Conditional Pseudo-F	Conditional *p*-Value
**Rainfall**	13	15.1	0.002	13	15.1	0.002
**Soil Moisture**	11.8	13.5	0.002	10	13	0.002
**Temperature mean**	11	12.4	0.002	5.4	7.5	0.002
**SOC**	9.6	10.8	0.002	1.1	1.6	0.15
**N**	3.6	3.7	0.012	2.1	3	0.048
**pH**	0.4	0.4	0.74	0.2	0.3	0.716

**Table 5:** Indicator species analysis showing taxa significantly associated with the climbing and independent growth behaviors of *O. baccatus*. The indicator value (IndVal%) and *p*-value are provided for each species for each behavior. Bolded species represent those with statistically significant associations (*p* < 0.05).

Species	Climbing		Independent	
	*p*-Value	IndVal%	*p*-Value	IndVal%
***Aerva javanica* (Burm.f.) Juss. ex Schult.**	0.2755	0.8121	0.0281	2.196
***Boerhavia repens* L.**	0.3373	0.6526	0.0649	2.053
***Haloxylon salicornicum* (Moq.) Bunge ex Boiss.**	0.0278	3.35	0.082	1.589
***Indigofera spinosa* Forssk.**	0.4318	0.4785	0.0363	2.183
***Lycium shawii* Roem. & Schult.**	0.0424	2.813	0.0124	2.824
***Panicum turgidum* Forssk.**	0.0368	3.016	0.0513	2.074
***Soda rosmarinus* (Bunge ex Boiss.) Akhani**	1	0	0.576	0.4911
***Vachellia flava* (Forssk.) Kyal. & Boatwr.**	0.1912	0.9571	0.0222	2.244
***Vachellia gerrardi* (Benth.) P.J.H.Hurter**	0.0408	2.726	0.0422	2.162
***Vachellia tortilis* (Forssk.) Galasso & Banfi**	0.0219	3.393	0.0626	2.053
***Zilla spinosa* (L.) Prantl**	0.0464	2.581	0.4153	0.9072
***Zygophyllum album* L.f.**	0.0148	3.582	0.9304	0.04775

## Data Availability

The original contributions presented in this study are included in the article. Further inquiries can be directed to the corresponding author.

## Supplementary Materials

The following supporting information can be downloaded at: https://www.mdpi.com/article/10.3390/plants14111696/s1, Table S1: Mean abundance and contribution of individual plant taxa to compositional dissimilarity among habitat types associated with *Ochradenus baccatus*, as identified by SIMPER analysis.
