# Supplementary material for: Vegetation Structure and Environmental Correlates of Climbing Behavior for Desert Shrub Ochradenus baccatus
Source: Plants (Basel). 2025 Jun 1;14(11):1696. doi: 10.3390/plants14111696 (PMC12157882; doi:10.3390/plants14111696)
Supplement: Supplementary file 1 [file plants-14-01696-s001.zip › plants-3646716-supplementary.pdf]

Supplementary Table S1. Mean abundance and contribution of individual plant taxa to compositional dissimilarity among habitat types associated with *Ochradenus baccatus*, as identified by SIMPER analysis.

Taxon	Av. dissim	Contrib. %	Cumulative %	Mean Plain land	Mean Sand dune	Mean Slope mountain	Mean Valley	Mean Rocky hill	Mean Plateau	Mean Sabkha	Mean Top Mountain	Mean Volcanic rocks
Haloxylon salicornicum (Moq.) Bunge ex Boiss.	2.449	3.053	3.053	7.53	10.7	0.875	3.94	10.5	0	0	0	0
Peganum harmala L.	2.112	2.633	5.686	6	11.2	3.88	2.8	22	0	0	6	0
Ochradenus baccatus Delile	2.05	2.556	8.242	9.68	6.67	6.75	7.16	7	2.25	5.25	11	12.7
Lycium shawii Roem. & Schult.	1.813	2.26	10.5	9.63	0.444	6.63	5.18	9.5	5.5	1	16	0
Vachellia gerrardi (Benth.) P.J.H.Hurter	1.605	2	12.5	6.05	1.22	7.5	4.66	7.5	4.5	1.25	12	0
Panicum turgidum Forssk.	1.486	1.853	14.36	6.84	3.11	5.5	5	0.5	4	2	8.75	0
Vachellia tortilis (Forssk.) Galasso & Banfi	1.398	1.743	16.1	6.58	0.556	4.88	5.8	2.5	5.75	1.25	8.25	3.33
Morettia parviflora Boiss.	1.291	1.61	17.71	1.95	1.78	1.13	3.12	0	3.5	2.5	0	0
Diplotaxis harra (Forssk.) Boiss.	1.281	1.597	19.3	2.05	1.22	2.13	2.78	0	5	1.75	0	0
Cornulaca monacantha Delile	1.224	1.526	20.83	1.63	1.78	0.375	2.7	0	6.75	1.75	0	0
Helianthemum lippii (L.) Dum.Cours.	1.216	1.516	22.35	2.63	0.556	1	2.66	0	5	0.5	0	0
Pulicaria jaubertii E.Gamal-Eldin	1.196	1.491	23.84	1.53	1.33	1.75	2.34	0	7	2.5	0	0
Lasiurus scindicus Henrard	1.179	1.47	25.31	1.42	0.667	1.13	3.02	0	4.5	2	0	0
Vachellia flava (Forssk.) Kyal. & Boatwr.	1.177	1.467	26.77	5.84	0	7.25	1.98	10	0	2.25	21.8	3.67
Zilla spinosa (L.) Prantl	1.166	1.453	28.23	2.26	2.22	2.5	3.76	0	6.25	0.75	0	4
Aerva javanica (Burm.f.) Juss. ex Schult.	1.153	1.437	29.66	5.68	0	7.88	1.8	11	0	1.75	17.8	5.67
Rhazya stricta Decne.	1.142	1.424	31.09	1.42	2.89	2.25	2.22	4	0	2.25	0	8
Suaeda vermiculata Forssk. ex J.F.Gmel.	1.126	1.404	32.49	1.74	3.67	0	2.22	0	0	8.75	0	0
Caroxylon imbricatum (Forssk.) Moq.	1.12	1.397	33.89	1.89	1.22	0.5	2.86	0	3.25	0.25	0	0

**Ramaliella musilii (Velen.) Zaika, Sukhor. & N.Kilian**	1.114	1.389	35.28	2.05	0	1.25	2.78	0	1.75	0.5	0	0
**Bassia muricata (L.) Asch.**	1.082	1.348	36.63	0.947	0.889	0.875	2.96	0	5	0.75	0	0
**Atriplex turcomanica (Moq.) Boiss.**	0.9689	1.208	37.84	3.42	3.33	0	0.86	0	0	0	0	0
**Zygophyllum album L.f.**	0.9474	1.181	39.02	0.368	0	0	4.94	0	0	0	0	0
**Artemisia monosperma Delile**	0.9396	1.171	40.19	1.95	4.22	0.625	1.06	0.5	0	4.5	0	0
**Periploca aphylla Decne.**	0.9373	1.168	41.36	1.16	1	0.75	2.12	0	5	1.25	0	0
**Calligonum comosum L'Hér.**	0.9371	1.168	42.52	1.26	5.78	0	0	0	0	10.3	0	0
**Indigofera spinosa Forssk.**	0.9313	1.161	43.68	5.47	0	6.75	1.68	9	0	0	23.3	0
**Boerhavia repens L.**	0.8884	1.107	44.79	6.47	0	7.5	1.58	11	0	0	15.5	0
**Solanum nigrum L.**	0.851	1.061	45.85	3.53	0	3.75	1.52	5.5	0	2.5	9.25	6.67
**Stipagrostis plumosa (L.) Munro ex T.Anderson**	0.8201	1.022	46.88	2.21	0.778	0.375	1.98	1.5	0	0	0	0
**Moltkiopsis ciliata (Forssk.) I.M.Johnst.**	0.8153	1.016	47.89	1.63	2	0	0	0	0	13	0	0
**Suaeda monoica Forssk. ex J.F.Gmel.**	0.8078	1.007	48.9	0.211	0	2.38	2.78	0	0	2	0	4.33
**Calotropis procera (Aiton) W.T.Aiton**	0.7907	0.9857	49.88	3.47	0	5.75	1.36	4	0	0.75	11	5.33
**Forsskaolea tenacissima L.**	0.7336	0.9145	50.8	2.84	0	3.25	1.28	10	0	2.25	10	3.67
**Cyperus conglomeratus Rottb.**	0.6811	0.849	51.65	0.211	3.33	0	0	0	0	11.8	0	0
**Echinops spinosissimus Turra**	0.6398	0.7976	52.45	3.63	0	5.63	0.88	8	0	0	15	0
**Retama raetam (Forssk.) Webb & Berthel.**	0.6289	0.784	53.23	1.32	1.33	1.25	0.94	1.5	0	0	0	0
**Halocnemum strobilaceum (Pall.) M.Bieb.**	0.6142	0.7656	54	1.58	4.89	0	0	0	0	1.5	0	0
**Cyperus longus L.**	0.598	0.7455	54.74	3.58	0	5.88	0.84	7	0	0	12	0
**Vachellia origena (Hunde) Kyal. & Boatwr.**	0.5892	0.7345	55.48	3.47	0	3.88	1.04	20	0	0	8.5	0

**Dodonaea viscosa Jacq.**	0.5802	0.7232	56.2	3.84	0	4.13	1	10.5	0	0	12.3	0
**Commiphora gileadensis (L.) C.Chr.**	0.5714	0.7123	56.91	0.211	0	2.13	0.88	0	0	2	0	7.67
**Centaurothamnus maximus (Forssk.) Wagenitz & Dittrich**	0.5625	0.7012	57.61	3.42	0	4.25	0.84	8	0	0	12	0
**Aloe armatissima Lavranos & Collen.**	0.5596	0.6975	58.31	2.79	0	4.25	0.92	8.5	0	0	13.3	0
**Cleome arabica L.**	0.5492	0.6847	58.99	0.421	1.78	0.75	1.36	2	0	0	0	0
**Soda rosmarinus (Bunge ex Boiss.) Akhani**	0.5439	0.6781	59.67	1.32	3.22	0	0	0	0	4.5	0	0
**Withania somnifera (L.) Dunal**	0.5387	0.6716	60.34	4.63	0	3.13	1	6	0	0	8.75	0
**Picris scabra Forssk.**	0.5339	0.6655	61.01	3.11	0	4	0.76	8	0	0	11.8	0
**Rhanterium epapposum Oliv.**	0.5257	0.6554	61.66	0.684	2.11	0	0	0	0	8.5	0	0
**Polycarpaea repens (Forssk.) Asch. & Schweinf.**	0.5211	0.6495	62.31	1.89	0.333	0	0.9	1.5	0	0	0	0
**Senna alexandrina Mill.**	0.5083	0.6337	62.95	0.158	0	1.88	0.52	0	0	1.25	0	9.67
**Psiadia punctulata Vatke**	0.5068	0.6317	63.58	3.42	0	2.63	0.98	9.5	0	0	11.8	0
**Eschenbachia stricta (Willd.) Raizada**	0.5061	0.6309	64.21	3.26	0	3.88	0.8	7	0	0	10.3	0
**Blepharis ciliaris (L.) B.L.Burtt**	0.5021	0.6259	64.84	3.68	0	4	0.62	12	0	0	8.25	0
**Rumex vesicarius L.**	0.4995	0.6226	65.46	0.368	0	2.38	0.64	0	0	0.75	0	6.67
**Pulicaria undulata (L.) C.A.Mey.**	0.4921	0.6135	66.07	3.68	0	2.88	1	9.5	0	0	9.25	0
**Heliotropium antiatlanticum Emb.**	0.4889	0.6095	66.68	0.0526	2.44	0	0	0	0	8.5	0	0
**Solanum incanum L.**	0.4872	0.6073	67.29	4.11	0	2.75	0.76	4	0	0	12	0
**Abutilon fruticosum Guill. & Perr.**	0.4835	0.6027	67.89	0.368	0	1.63	0.5	0	0	2	0	7.33
**Cymbopogon schoenanthus (L.) Spreng.**	0.4813	0.5999	68.49	3.58	0	4.25	0.82	11	0	0	6.25	0

**Pulicaria incisa (Lam.) DC.**	0.4785	0.5965	69.09	0.316	0	1.5	0.62	0	0	2	0	7
**Sesbania sesban (L.) Merr.**	0.4731	0.5897	69.68	0.368	0	1.88	0.48	0	0	1.75	0	6.67
**Artemisia sieberi Besser**	0.4686	0.5842	70.26	0.211	2.67	0.25	0.62	3	0	0	0	0
**Piptatherum holciforme (M.Bieb.) Roem. & Schult.**	0.4582	0.5712	70.83	3.37	0	4	0.88	4.5	0	0	8.75	0
**Otostegia fruticosa (Forssk.) Schweinf. ex Penz.**	0.457	0.5697	71.4	3.89	0	2.25	0.74	3.5	0	0	11	0
**Trichodesma trichodesmoides (Bunge) Gürke**	0.4566	0.5692	71.97	4	0	3.25	0.84	0.5	0	0	11.3	0
**Juniperus procera Hochst. ex Endl.**	0.4525	0.564	72.54	3.95	0	4.13	0.88	0	0	0	8	0
**Lavandula dentata L.**	0.4508	0.562	73.1	3.47	0	3.25	0.76	7.5	0	0	9	0
**Cyperus bulbosus Vahl**	0.4476	0.558	73.66	0	0	0	2.36	0	0	0	0	0
**Rumex nervosus Vahl**	0.4431	0.5524	74.21	3.32	0	2.5	0.98	9.5	0	0	8	0
**Euphorbia schimperiana Scheele**	0.4388	0.547	74.76	3.58	0	2.5	0.76	5	0	0	10	0
**Eragrostis curvula (Schrad.) Nees**	0.4374	0.5453	75.3	3.74	0	3.38	0.8	5	0	0	7.75	0
**Teucrium yemense Deflers**	0.4371	0.5449	75.85	3.47	0	3.63	0.88	0	0	0	10.5	0
**Combretum molle R.Br. ex G.Don**	0.4352	0.5425	76.39	3.05	0	3.75	0.94	0	0	0	11.5	0
**Verbesina encelioides (Cav.) Benth. & Hook.f. ex A.Gray**	0.4351	0.5424	76.93	3.89	0	1.88	0.86	2.5	0	0	11	0
**Ficus vasta Forssk.**	0.435	0.5422	77.47	3.95	0	3.63	0.82	0	0	0	9.25	0
**Datura stramonium L.**	0.4344	0.5416	78.02	4	0	2.75	0.82	0.5	0	0	10.3	0
**Phragmites australis (Cav.) Trin. ex Steud.**	0.4291	0.535	78.55	0.0526	0	1.38	0.66	0	0	0.5	0	7.67
**Onopordum heteracanthum C.A.Mey.**	0.4245	0.5292	79.08	4.16	0	2	0.94	0	0	0	9.5	0

**Datura innoxia Mill.**	0.4236	0.5281	79.61	0.316	0	1.13	0.58	0	0	1.5	0	6.33
**Marrubium vulgare L**	0.423	0.5273	80.13	3.89	0	2.75	0.88	0	0	0	9.25	0
**Lavandula pubescens Decne.**	0.422	0.526	80.66	3.21	0	3.38	0.86	2.5	0	0	9.5	0
**Searsia retinorrhoea (Steud. ex Oliv.) Moffett**	0.4214	0.5253	81.19	3.32	0	3.5	1.12	0	0	0	8.5	0
**Buddleja polystachya Fresen.**	0.4109	0.5122	81.7	3.58	0	3.5	0.74	0	0	0	9	0
**Aristida funiculata Trin. & Rupr.**	0.4089	0.5097	82.21	3.53	0	3.63	1.02	1	0	0	7	0
**Pistacia khinjuk Stocks**	0.4036	0.5031	82.71	3.79	0	3.25	0.8	0	0	0	8	0
**Cenchrus setaceus (Forssk.) Morrone**	0.4015	0.5005	83.21	3.26	0	2.75	0.6	0	0	0	11.5	0
**Hypericum revolutum Vahl**	0.401	0.4998	83.71	3.53	0	3.88	0.8	0	0	0	7.5	0
**Adenium obesum (Forssk.) Roem. & Schult.**	0.3897	0.4858	84.2	3.11	0	3.25	1	0	0	0	8.5	0
**Solenostemma arghel (Delile) Hayne**	0.3896	0.4857	84.68	0.316	0	0.875	0.62	0	0	1.5	0	5.33
**Prosopis juliflora**	0.3866	0.482	85.16	0.211	0	0.875	0.68	0	0	1	0	6
**Clutia jaubertiana Müll.Arg.**	0.3833	0.4779	85.64	3.26	0	3.38	0.88	0	0	0	7	0
**Maesa lanceolata Forssk.**	0.3824	0.4766	86.12	2.89	0	3.25	0.92	0	0	0	8.5	0
**Opuntia ficus-indica (L.) Mill.**	0.3802	0.4739	86.59	3.42	0	3	0.82	0	0	0	7.25	0
**Suaeda aegyptiaca (Hasselq.) Zohary**	0.3802	0.4739	87.07	0.158	0	1.75	0.64	0	0	0.75	0	4.33
**Olea europaea L.**	0.3799	0.4736	87.54	3	0	2.75	1.12	0	0	0	8.25	0
**Artemisia judaica L.**	0.3747	0.4671	88.01	2.21	0.444	0.25	0.64	0.5	0	0	0	0
**Dianthus uniflorus Forssk.**	0.3708	0.4622	88.47	3.26	0	3.13	0.82	0	0	0	6.75	0
**Erigeron incanus Vahl**	0.3703	0.4616	88.93	3.63	0	2.88	0.68	0	0	0	6.75	0
**Ficus salicifolia Vahl**	0.3688	0.4598	89.39	3.58	0	2.63	0.82	0	0	0	7	0

**Pupalia lappacea (L.) Juss.**	0.3656	0.4558	89.85	2.84	0	3.13	0.84	0	0	0	8.25	0
**Euryops arabicus Steud. ex Jaub. & Spach**	0.3631	0.4526	90.3	3.05	0	2.13	0.76	5	0	0	6.75	0
**Zygophyllum coccineum L.**	0.355	0.4425	90.74	0.211	0	1.13	0.52	0	0	1.25	0	5
**Ziziphus spina-christi (L.) Desf.**	0.3506	0.437	91.18	1.11	0.333	0.5	0.38	0	0	0.5	0	5
**Vachellia tortilis subsp. raddiana (Savi) Kyal. & Boatwr.**	0.344	0.4288	91.61	0.316	0.111	0.375	1.28	0.5	0	0	0	0
**Tribulus macropterus Boiss.**	0.3413	0.4255	92.03	0.263	0	1.75	0.36	0	0	1.25	0	3.67
**Maerua crassifolia Forssk.**	0.3108	0.3875	92.42	0.316	0.111	0	1.24	0.5	0	0	0	0
**Phoenix dactylifera L.**	0.307	0.3828	92.8	0.421	0	0	0.7	0	0	0.25	0	4
**Aeluropus lagopoides (L.) Thwaites**	0.3066	0.3822	93.19	0.579	0	0	1.18	0	0	0	0	0
**Trichodesma africanum (L.) Sm.**	0.2952	0.368	93.55	0.211	0	1	0.52	0	0	0.75	0	3.33
**Nitraria retusa (Forssk.) Asch.**	0.2897	0.3612	93.91	0.474	0	0	1.18	0	0	0	0	0
**Heliotropium bacciferum Forssk.**	0.2884	0.3595	94.27	0.421	0	0	1.16	0	0	0	0	0
**Zygophyllum paulayanum (J.Wagner & Vierh.)**	0.2853	0.3557	94.63	0.158	0	1.38	0.68	0	0	1.25	0	0
**Vachellia oerfota (Forssk.) Kyal. & Boatwr.**	0.2571	0.3204	94.95	0.0526	0	0.875	0.54	0	0	0	0	3.67
**Tamarix aphylla (L.) H.Karst.**	0.2561	0.3192	95.27	0.368	0	1	0.22	0	0	0.5	0	3.33
**Zygophyllum simplex L.**	0.2465	0.3073	95.58	0.0526	0	1.13	0.28	0	0	1	0	3.33
**Pergularia tomentosa L.**	0.2441	0.3043	95.88	0.211	0.444	0.25	0.82	0.5	0	0	0	0
**Astragalus dactylocarpus Boiss.**	0.237	0.2955	96.18	0.316	3.33	0	0	0	0	0	0	0
**Tamarix nilotica (Ehrenb.) Bunge**	0.2348	0.2927	96.47	0.211	0.444	0	0.88	0.5	0	0	0	0

**Ficus palmata Forssk.**	0.2299	0.2866	96.76	0.684	0	0	0.7	0	0	0	0	0
**Astragalus asterias Steven**	0.2172	0.2708	97.03	0.789	2.44	0	0	0	0	0	0	0
**Iphiona scabra DC. ex Decne.**	0.215	0.2681	97.3	0.368	0.556	0.375	0.24	1.5	0	0	0	0
**Astragalus spinosus (Forssk.) Muschl.**	0.2085	0.2599	97.56	0.368	0.444	1	0.06	0.5	0	0	0	0
**Leptadenia pyrotechnica (Forssk.) Decne.**	0.2056	0.2563	97.81	0.105	0.556	0	0.72	0	0	0	0	0
**Hyoscyamus muticus L.**	0.1857	0.2315	98.04	0.474	0.222	0.25	0.3	1	0	0	0	0
**Schismus barbatus (L.) Thell.**	0.1857	0.2315	98.27	1.37	1.22	0	0	0	0	0	0	0
**Senegalia asak (Forssk.) Kyal. & Boatwr.**	0.1703	0.2124	98.49	0.0526	0	0.75	0.14	0	0	1	0	2.33
**Hyphaene thebaica (L.) Mart.**	0.153	0.1907	98.68	0.158	0.222	0	0.48	1	0	0	0	0
**Cenchrus divisus (J.F.Gmel.) Verloove, Govaerts & Buttler**	0.1464	0.1825	98.86	1.84	0	0	0	0	0	0	0	0
**Savignya parviflora (Delile) Webb**	0.1313	0.1637	99.02	0.684	1.22	0	0	0	0	0	0	0
**Achillea fragrantissima (Forssk.) Sch.Bip.**	0.1297	0.1617	99.19	0.0526	0.333	0.75	0.06	0.5	0	0	0	0
**Cleome droserifolia (Forssk.) Delile**	0.1172	0.1461	99.33	0	0	0.25	0.46	0	0	0	0	0
**Capparis decidua (Forssk.) Edgew.**	0.1103	0.1375	99.47	0.211	0.111	0.25	0.24	0	0	0	0	0
**Gomphocarpus sinaicus Boiss.**	0.09388	0.117	99.59	0.0526	0.444	0.25	0.06	0.5	0	0	0	0
**Coincya tournefortii (Gouan) Alcaraz, T.E.Díaz, Rivas Mart. & Sánchez-Gómez**	0.08499	0.106	99.69	0	1.33	0	0	0	0	0	0	0
**Calendula tripterocarpa Rupr.**	0.07791	0.09713	99.79	0	1.22	0	0	0	0	0	0	0
**Searsia tripartita (Ucria) Moffett**	0.06795	0.08471	99.87	0.0526	0.333	0.125	0.04	0.5	0	0	0	0
**Matricaria aurea (Loefl.) Sch.Bip.**	0.06375	0.07947	99.95	0	1	0	0	0	0	0	0	0

**Mesembryanthemum cryptanthum Hook.f.**	0.037<br>65	0.046<br>94	100	0.474	0	0	0	0	0	0	0	0
